# Biodegradation of Dimethyl Phthalate by Freshwater Unicellular Cyanobacteria

**DOI:** 10.1155/2016/5178697

**Published:** 2016-12-19

**Authors:** Xiaohui Zhang, Lincong Liu, Siping Zhang, Yan Pan, Jing Li, Hongwei Pan, Shiguo Xu, Feng Luo

**Affiliations:** ^1^Research Center of Bioenergy and Bioremediation, College of Resource and Environment, Southwest University, Chongqing 400715, China; ^2^Institute of Environment and Ecology, Shandong Normal University, 88 Wenhua Donglu, Jinan, Shandong 250014, China

## Abstract

The biodegradation characteristics of dimethyl phthalate (DMP) by three freshwater unicellular organisms were investigated in this study. The findings revealed that all the organisms were capable of metabolizing DMP; among them,* Cyanothece* sp. PCC7822 achieved the highest degradation efficiency. Lower concentration of DMP supported the growth of the Cyanobacteria; however, with the increase of DMP concentration growth of Cyanobacteria was inhibited remarkably. Phthalic acid (PA) was detected to be an intermediate degradation product of DMP and accumulated in the culture solution. The optimal initial pH value for the degradation was detected to be 9.0, which mitigated the decrease of pH resulting from the production of PA. The optimum temperature for DMP degradation of the three species of organisms is 30°C. After 72 hours' incubation, no more than 11.8% of the residual of DMP aggregated in Cyanobacteria cells while majority of DMP remained in the medium. Moreover, esterase was induced by DMP and the activity kept increasing during the degradation process. This suggested that esterase could assist in the degradation of DMP.

## 1. Introduction

Phthalic acid esters (PAEs) are a class of refractory organic plasticizer compounds that are widely utilized as additives and plasticizers in a variety of polymeric products, including paints, cosmetics, and flexible plastics, such as plastic toys and containers [1–3]. Consequently, they are found spreading ubiquitously at high concentrations in terrestrial and aquatic ecosystems [4–6]. In addition, PAEs are endocrine-disrupting pollutants and can show adverse effects on the environment and human health, inducing hepatic peroxisome proliferation, hormonal disorders, reproductive toxicity, and carcinogenicity [7, 8]. They are classified as priority pollutant in the United States, the European Union, and China [6, 9, 10]. Dimethyl phthalate (DMP) is one kind of PAEs and the solubility in water is one of the highest among all the PAEs, reaching 4500 mg L^−1^ at 25°C; inevitably, it could accumulate in the aquatic systems. Although DMP has only a moderate toxicity, its metabolic intermediate mono-methylphthalate (MMP) is not only toxic but also an endocrine disruptor and may interfere with the development and reproductive system of animals, or even human, by reducing testosterone production and decreased sperm counts [11]. Subsequently, MMP can be further degraded into phthalic acid (PA) and finally CO_2_ and H_2_O [12]. Due to the wide distribution, persistence, and difficulty in removal of PAEs, treatment approaches for PAEs-contaminated wastewater have attracted many researchers' attention [13–15]. Different methods for removing PAEs from the environment include physical, chemical, and biological treatments, advanced oxidation processes, and combinations of these techniques [16–20]. Unfortunately, these methods are limited in application because of the high cost. In recent years, a number of studies have been carried out to evaluate the degradation of PAEs by bacteria in terrestrial and aquatic ecosystems [12, 21]. Cyanobacteria are a class of autotrophic microorganisms capable of photosynthesis. Cyanobacteria have a high photosynthetic efficiency (upwards of 10%), significantly better than land plants (~3-4% maximum efficiency) [22, 23]. As the primary producers, Cyanobacteria and microalgae support more than half of the global primary production in the aquatic food web. Pollutants can enter aquatic food chains through bioaccumulation of Cyanobacteria and microalgae, so their response will certainly affect upper tropic levels. Moreover, it has been well known that microorganism degradation is a vital process affecting the environmental fate of PAEs [20, 24] In addition to producing oxygen to meet the needs of heterotrophic bacteria and subsequently stimulate the activities of the bacteria degrading organic pollutants, Cyanobacteria and microalgae are also capable of degrading organic pollutants directly, such as phenolics, polycyclic aromatic hydrocarbons, pesticides, petroleum, and PAEs [25]. However, most of those studies have focused on determining either optimizing conditions of PAEs or the degradation efficiency in some groups of organisms. Comprehensive degradation characters of aquatic prokaryotes have not yet been elucidated. Moreover, genome sequences and genetic manipulation techniques are insufficient for the Cyanobacteria used in the previous studies.

Therefore, characteristics of DMP biodegradation of three model freshwater unicellular Cyanobacteria, including two nonnitrogen fixing Cyanobacteria* Syenchocystis *sp. PCC6803,* Synechococcus* sp. PCC7942 and a diazotrophic one,* Cyanothece* sp. PCC7822, were illuminated. Most importantly, the three photoautotrophic Cyanobacteria have the advantages that their genome sequences are available and all the three species can be manipulated by genetic engineering [26–28]. The objective of this study is to investigate the effect of DMP on the growth of Cyanobacteria and to characterize the DMP biodegradation of the three Cyanobacteria. Furthermore, the key enzyme activity was assayed. This study provides a novel idea to apply these three model Cyanobacteria to the bioremediation of PAEs and supplies basic knowledge for studying the mechanism in the PAEs degradation in molecular level in the near future.

## 2. Materials and Methods

### 2.1. Chemicals and Cyanobacteria

Dimethyl phthalate (DMP), mono-methylphthalate (MMP), phthalic acid (PA), and 4-nitrophenyl acetate were purchased from Sigma-Aldrich (Steinem, Germany). Bio-Rad Protein Assay Kit II was purchased from Bio-Rad Company (Hercules, Calif. USA). Three species of freshwater unicellular Cyanobacteria were provided by Purdue University, West Lafayette, USA, including two nonnitrogen fixing Cyanobacteria* Synechocystis *sp. PCC6803,* Synechococcus* sp. PCC7942, and a diazotrophic* Cyanothece* sp. PCC7822. 100 mL of Cyanobacteria cells was cultivated in sterilized BG11 medium in 250 mL flasks in the laboratory at 25 ± 1°C under a 16 : 8 light : dark cycle provided by cool white fluorescent tubes at a light intensity of 50 *μ*mol photons m^−2^ s^−1^. Prior to being used for the experiments, the cells were maintained in the exponential growth phase by repeated inoculation into fresh medium. All samples were prepared in triplicate and incubated using a rotary shaker operated at 100 rpm.

### 2.2. Effect of DMP on the Growth of Cyanobacteria

To evaluate the effect of DMP on the growth of Cyanobacteria, the growth curves of three Cyanobacteria under a variety of concentrations of DMP were investigated. Cyanobacteria cells in the middle exponential phase of growth were harvested by centrifugation (10000 g, 10 min). The collected cells were washed twice with BG11 medium for experiments. A stock solution of DMP 4000 mg L^−1^ in BG11 medium was prepared. Different concentrations of DMP 0, 20, 50, 100, 200, and 500 mg L^−1^ in the culture were obtained by diluting the stock solution with BG11 medium. Growth was determined every 24 h for up to 120 hours, based on the absorbance at 730 nm (OD_730_) using a UV-vis spectrophotometer Shimadzu UV2600 (Shimadzu, Kyoto, Japan). The initial OD_730_ of the Cyanobacteria culture were set around 0.10 and cells were cultivated at the same condition as the preculture. 2 mL cells were taken every 24 h for sampling at time points of 0, 24, 48, 72, 96, and 120 h.

### 2.3. Biodegradation of DMP by Cyanobacteria

The stock solution of DMP was diluted with sterilized BG11 medium to reach DMP of 20 and 50 mg L^−1^. The final cell density of each Cyanobacteria species was measuring the optical density at OD_730_ around 0.15. Supernatant separated from triplicate flasks of each culture every 24 hours was utilized for the DMP measurement. The DMP extraction treatment included centrifuging the culture for 10,000 g for 10 min and filtrating the supernatant through a 0.45 *μ*m pore-size filter for HPLC assay.

### 2.4. Effect of Temperature on the DMP Biodegradation

A series of temperatures 15°C, 25°C, 30°C, 35°C, and 40°C were set to compare the degradation efficiency of the three species of Cyanobacteria. The initial OD_730_ of the Cyanobacteria culture were set around 0.10 and cells were cultivated at the same condition as the preculture. Cultures with 50 mg L^−1^ of DMP were inoculated in growth chambers (RDN-400C-4, Dongnan Co. Ltd., China) provided with LED light intensity of 50 *μ*mol photons m^−2^ s^−1^ under a 16 : 8 light : dark cycle. After 96 h, the DMP degradation efficiency was calculated.

### 2.5. Effect of Initial pH on the Biodegradation

The pH values of the culture medium of* Cyanothece* sp. PCC7822 with 20 and 50 mg L^−1^ DMP were measured every 24 h for 120 hours. 10 mL culture samples were taken for pH measurement. Simultaneously, the PA accumulation in same cultures was investigated and PA concentration was assayed by using HPLC methods. A series of initial pH of 6.0, 7.0, 8.0, 9.0, and 10.0 in the BG11 medium amended with 50 mg L^−1^ DMP were adjusted by HCl and NaOH. Cells with an intensity of OD_730_ 0.15 were inoculated in the medium for the study, DMP degradation efficiency was calculated under different initial pH conditions after 72 h.

### 2.6. High Performance Liquid Chromatography (HPLC) Analysis

For the analysis of extracellular residual DMP, MMP, and PA in the solution, 2 mL of Cyanobacteria solution was centrifuged (10,000 rpm, 10 min). The supernatant was collected and filtered through a 0.45 *μ*m pore-size filter for analysis in HPLC. For the DMP concentrations in vivo, cells were harvested from 20 mL of culture medium by centrifugation at 10,000 g for 10 min. Then the pellet was disrupted by grinding with liquid nitrogen and suspended with 2 mL dichloromethane at 5000 rpm for 10 min. The solution was centrifuged at 4000 rpm for 10 min. After discarding of the water phase, the dichloromethane layer was concentrated to a final volume of 0.5 mL. An analytical high performance liquid chromatography LC-20AD (Shimadzu, Kyoto, Japan) was used to analyze the DMP and its metabolites DMP, MMP, and PA. Samples were analyzed by a capillary column InertSustain C18 (4.6 mm × 150 mm × 5 *μ*m); The mobile phase used was methanol—0.1% phosphoric acid solution (50 : 50, v/v) with a flow rate of 1.0 mL/min. The sample volume of 20 *μ*L was injected into an oven temperature 30°C, with a UV detector, and detecting wavelength was set at 226 nm. The retention times of DMP MMP and PA were identified according to the retention time of the reference standards.

### 2.7. Esterase Activity Assay

Cyanobacterial cells were cultivated with 50 mg L^−1^ DMP or without DMP as a control. Cells were harvested by centrifugation at 10,000 g for 10 min at 4°C, after three times washing with 50 mM potassium phosphate (pH 7.0), the pellet was disrupted by grinding with liquid nitrogen and suspended in the same buffer followed by centrifuge for 10 mins at 4°C, and supernatant was then subject to the enzyme assay, as described previously [29, 30] with some modifications. The reaction mixture included 50 mM of potassium phosphate buffer at pH 7.0 with an appropriate amount of cell-free enzyme extract and 100 *μ*L of 25 mM 4-nitrophenyl acetate diluted to a total volume of 1 mL at 25°C. The protein content was estimated using the Bio-Rad Protein Assay Kit II using BSA as the standard according to the manufacturer's instruction. The reaction was stopped by adding 50 *μ*L of 2 N HCL. The optical density at 400 nm of reaction mixture was measured using a UV-vis spectrophotometer UV2600 (Shimadzu, Kyoto, Japan). A blank was measured using only buffer. The increase of the absorbance at 400 nm implies the increase of 4-nitrophenolate as a product of the reaction resulted from the hydrolysis of 4-nitrophenyl acetate. The enzyme activity was presented in the unit of U mL^−1^ as in the previous studies [30]. Experimental data was analyzed with SPSS software (version 17.0).

## 3. Results and Discussion

### 3.1. Correlations between DMP Concentration and Cyanobacteria Growth

**Figure 1:**
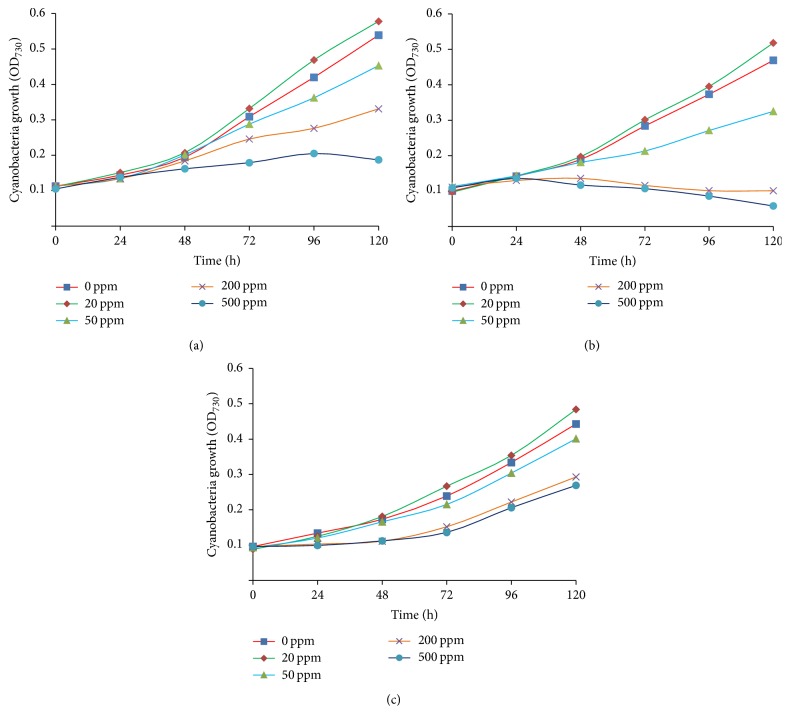
Effect of different concentration of DMP on the growth of three Cyanobacteria. (a)* Synechocystis *sp. PCC6803 (6803), (b)* Synechococcus* sp. PCC7942 (7942), and (c)* Cyanothece* sp. PCC7822 (7822).

Phthalic acid esters are endocrine-disrupting compounds, which have been shown to reduce the diversity of microbial communities and decline crop quality [31], and are toxic to most aquatic organisms including algae [32]. Higher concentration of DMP inhibits the growth rate, while lower concentration can induce the growth of Cyanobacteria or algae [33, 34]. However, it was also reported that, even at as high as 200 mg L^−1^, PAEs shows a promotive effect on the growth of Cyanobacteria [30]. In this study, DMP was introduced to BG11 medium at concentrations of 0, 20, 50, 200, and 500 mg L^−1^, and its effects on the growth of three Cyanobacteria were evaluated over 120 hours. A growth-stimulative effect of DMP was found only at the concentration of 20 mg L^−1^, but higher concentration of DMP (≥50 mg L^−1^) revealed growth inhibition activity with increasing concentrations of DMP during the study period 120 h.  Figure 1 shows the typical growth patterns of all three Cyanobacteria in the absence or presence of each concentration of DMP. However, with the increase of the concentration of DMP, three Cyanobacteria showed different degree of growth inhibition and all showed serious growth deduction at higher concentration. The growth of* Synechocystis* was inhibited completely at the DMP concentration of 500 mg L^−1^ (Figure 1(a)).* Synechococcus* was more sensitive to higher concentration of DMP; 200 mg L^−1^ of DMP suppressed the growth entirely (Figure 1(b)). Among them,* Cyanothece* sp. 7822 showed the most tolerance to higher concentration of DMP; its growth maintained to continue under the highest concentration applied (Figure 1(c)). Different Cyanobacteria showed different tolerance to the DMP; the reason could be due to their surface composition and surface area exposed to the DMP. For instance,* Cyanothece* sp. PCC7822 has a thicker layer of extracellular polysaccharide, which may aid the cells to tolerate the toxicity in the environments. The intracellular and extracellular enzymes involved in the degradation of DMP are also important in supporting the cells that survive in the stress conditions.

Additionally, the maximum growth rate of the three Cyanobacteria was observed in the presence of 20 mg L^−1^ DMP; this suggests that low concentration DMP are taken up and degraded by the cyanobacterial cells and finally used as a carbon source. These results revealed a significant difference between the samples incubated in different concentrations of DMP. A positive correlation was observed between the 20 mg L^−1^ DMP and growth rate in all three species and a negative correlation between growth and high DMP concentration.

### 3.2. Biodegradation of DMP

**Figure 2:**
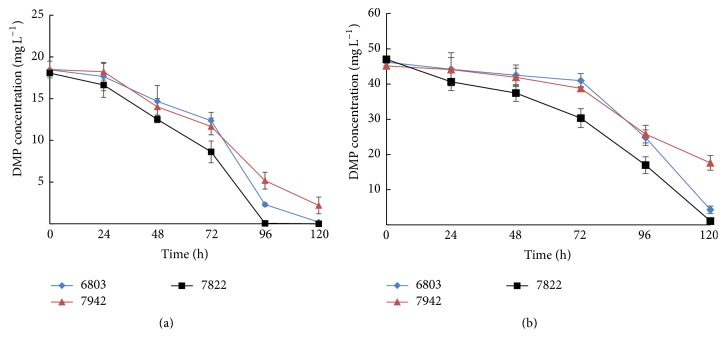
Change of the DMP concentrations of the culture medium over time by* Synechocystis *sp. PCC6803 (6803),* Synechococcus* sp. PCC7942 (7942), and* Cyanothece* sp. PCC7822 (7822). With an initial DMP concentration around 20 mg L^−1^ (a) and 50 mg L^−1^ (b).

The biodegradation tendency of DMP was studied by incubating cyanobacterial cultures with 20 and 50 mg L^−1^ of DMP and then measuring the DMP that remained in the culture medium every 24 hours during the incubation process, as shown in Figure 2. There was a similar decreasing trend of DMP concentration over time in all three species. However, DMP degraded more rapidly in cultures incubated with* Cyanothece* than in the other two cultures. Apparently, there was no trace amount of DMP detected in* Cyanothece* culture after incubation for 96 hours with an initial concentration of 20 mg L^−1^ and 120 hours with 50 mg L^−1^ of DMP. The time course of biodegradation of DMP was similar in the three Cyanobacteria and the degradation tendency of the three Cyanobacteria is equivalent to that of the microalgae* D. tertiolecta* [33]. The degradation rate was slow at the beginning and gradually increased with the incubation time; eventually it reached highest at the time span from 72 h to 96 h. In the cultures with initial concentration of 20 mg L^−1^ the degradation rate was 0.357 mg L^−1^ h^−1^ for* Cyanothece* and 0.271 mg L^−1^ h^−1^ for* Synechococcus*. In the cultures with initial concentration of 50 mg L^−1^, although the* Cyanothece* completed the degradation earlier,* Synechocystis* showed the highest degradation rate 0.6746 mg L^−1^ h^−1^, slightly higher than* Cyanothece* 0.5567 mg L^−1 ^h^−1^.

During the degradation of DMP,* Cyanothece* displayed the ability to tolerate and degrade the DMP more efficiently than the other two species of Cyanobacteria.* Synechocystis* showed a moderate biodegradation ability and* Synechococcus* showed the lowest. The highest degradation rate of* Cyanothece* is 8.75 mg L^−1^ d^−1^ in the initial DMP concentration 20 mg L^−1^ and 15.85 mg L^−1^ d^−1^ in the initial concentration 50 mg L^−1^, which is comparable to that of* Dunaliella* exhibited biodegradation rates of 11.3–30.5 mg L^−1^ d^−1^ for 100–300 mg L^−1^ of DMP [33].* Chlorella pyrenoidosa* showed a lower biodegradation rate with average biodegradation rates between 2.1 and 13.4 mg L^−1^ d^−1^ being observed for DMP [35]. During the biodegradation of DMP, organisms such as the green alga* Chlorella vulgaris* [36] and bacteria [37, 38] generally follow a first-order reaction model, while some other green algae such as* Dunaliella tertiolecta* [33] follow a second-order reaction pattern. The results of our present study showed the pattern of DMP degradation of Cyanobacteria is more likely to be a second-order reaction model (Figure 2). The degradation products were subsequently analyzed with HPLC. DMP and its intermediate degradation metabolites MMP and PA were shown in Figure  S1 (see Supplementary Material available online at http://dx.doi.org/10.1155/2016/5178697). The DMP was detected at a retention time of 8.278 min and the compound detected at a retention time of 4.567 min was identical to that of authentic MMP and 3.098 min was identical to that of PA in the degradation products.

### 3.3. Effect of Temperature on the DMP Biodegradation

**Figure 3:**
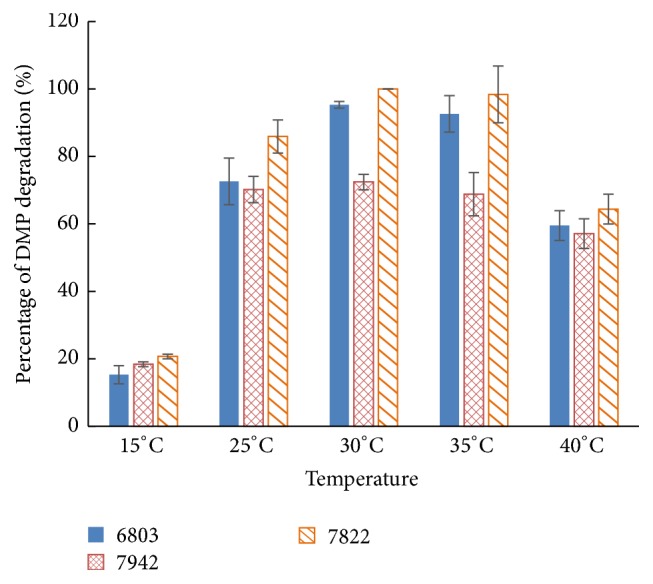
The influence of temperature on the efficiency of DMP biodegradation by* Synechocystis *sp. PCC6803 (6803),* Synechococcus* sp. PCC7942 (7942), and* Cyanothece* sp. PCC7822 (7822).

Similar as the other microorganisms, the DMP degradation of Cyanobacteria is influenced by the factor of temperature. The data in Figure 3 showed the dependence of the degradation efficiency on the temperature. After 96 h incubation, almost all the DMP of 50 mg L^−1^ in the cultures was degraded at 30°C and 35°C. The degradation efficiency of the three species of Cyanobacteria at 30°C is slightly higher than that at 35°C and* Cyanothece* sp. PCC7822 finished the degradation of DMP completely. At 15°C, three Cyanobacteria performed the lowest degradation ability, less than 20%. Surprisingly, at 40°C, Cyanobacteria still remained to have active degradation abilities; even though the cells appeared yellowish, more than 60% of the DMP was degraded, comparable to 25°C. In the previous studies, no matter bacteria or microalgae, experimental temperatures were set at around 25°C [11, 30, 35–37], taking the environmental temperature into consideration. However, at 25°C, the degradation of DMP of the three Cyanobacteria is about 50–60% of that at 30°C. Optimum temperature for the three species of Cyanobacteria to degrade DMP is 30°C and at this temperature, cells condition appears healthy.

### 3.4. Accumulation of PA and Change of pH

**Figure 4:**
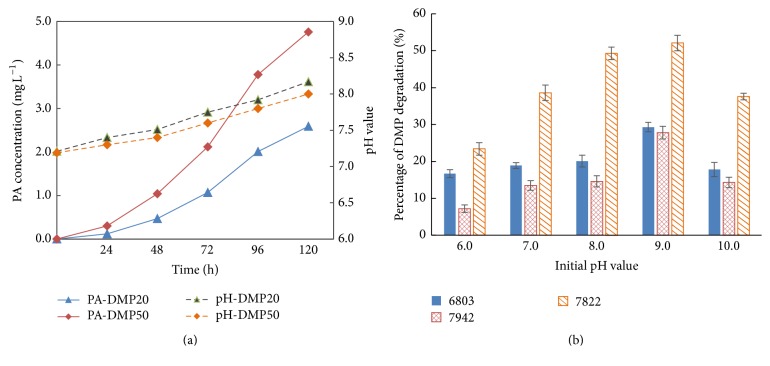
The process of PA accumulation and pH change of the* Cyanothece* sp. PCC7822 (a) and the influence of initial pH on the efficiency of DMP biodegradation by* Synechocystis *sp. PCC6803 (6803),* Synechococcus* sp. PCC7942 (7942), and* Cyanothece* sp. PCC7822 (7822) (b).

**Figure 5:**
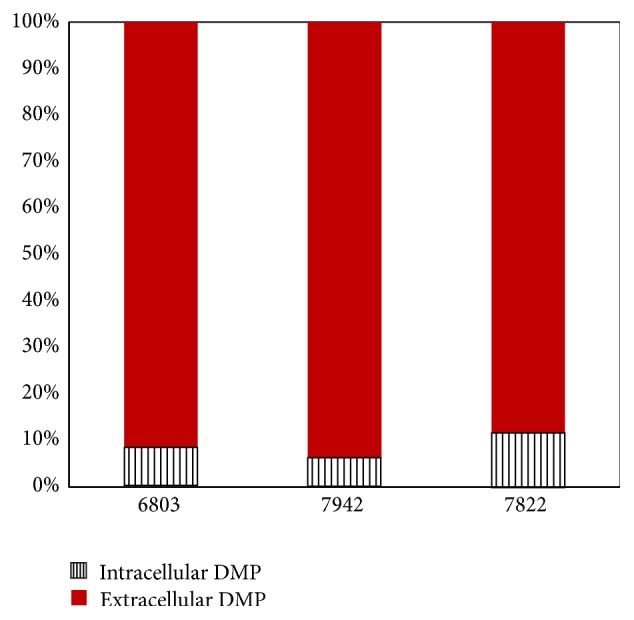
Percentages of residual DMP distributed in* Synechocystis *sp. PCC6803 (6803),* Synechococcus* sp. PCC7942 (7942), and* Cyanothece* sp. PCC7822 (7822) cells (intracellular DMP), and in the medium (extracellular DMP) incubated with 50 mg L^−1^ DMP for 72 hours.

During the process of incubation, DMP was degraded by* Cyanothece* and the PA as an intermediate product accumulated in culture medium. The PA concentrations gradually increased, which is in direct proportion to initial DMP concentration. The highest net production of PA was also between hours 72 and 96 during the degradation (Figure 4(a)). Within the most efficient period, net PA production speed was 0.039 and 0.069 mg L^−1 ^h^−1^ with DMP initial concentration of 20 and 50 mg L^−1^. Distribution patterns of DMP in three Cyanobacteria culture at 72 hour are shown in Figure 5. The residual DMP mainly presents in medium (extracellular) and no more than 11.8% was in* Cyanothece* cell (intracellular) phase. In* Synechocystis* the extracellular DMP occupied only 8.5% and for* Synechococcus* only 6.3%.

The accumulation of PA resulted in the deduction of the pH. As shown in Figure 4(a) the pH values of the culture with 20 mg L^−1^ DMP are always higher than that with 50 mg L^−1^ DMP. The initial pH of BG11 medium is around 7.2. After the 120 hours' incubation, pH values increased 0.94 and 0.81, respectively. To investigate the optimum initial pH for the DMP biodegradation, a variety of pH values were set ranging from 6.0 to 10.0 and the Cyanobacteria showed different efficiency in the DMP degradation. In the culture with initial pH 6.0, three species of Cyanobacteria showed the lowest degradation functions. Initial pH 9.0 is the optimum value for DMP degradation (Figure 4(b)). Generally speaking, during growth of Cyanobacteria pH values of the cultures increase gradually due to the assimilation of NaHCO_3_ and NaNO_3_ and as carbon and nitrogen source, and alkaline medium is more suitable for the Cyanobacteria growth; however, with the accumulation of PA, the pH increase was deducted. The inhibition of Cyanobacteria growth showed in Figure 1 is not only due to the toxicity of DMP but also caused by its intermediate metabolites MMP and PA (Figure 4).

### 3.5. Enzyme Activity for DMP Degradation

The biodegradation efficiency for DMP was evaluated in vitro using enzyme extracts from Cyanobacteria from the culture amended with or without DMP. As shown in Table 1, the esterase activities were significantly lower in all three Cyanobacteria grown in the absence of DMP. However, an obvious elevation was observed when cells were grown in the presence of DMP. As shown in Table 1, after 72 hours' incubation, the esterase activities of Cyanobacteria without DMP were equivalent to that of the cells just being introduced with DMP. However, a drastic elevation in enzyme activity was observed with the increase of incubation time when cells were in the presence of DMP. From 0 h to 72 h after being exposed to DMP, the esterase activity increased 14.5–160.0 times. The activities at 24 hour were only 28.1–31.5% of the activity at 72 hours. Similar phenomena were observed in all three tested organisms and a slightly higher enzyme activity was observed for* Cyanothece* sp. PCC 7822.

The ester hydrolyzing enzymes (esterases) are a specified group of enzymes (EC 3.1.1.2), which are very vital for the primary attack of xenobiotics, including phthalic derivatives. Microbial degradation of PAEs is initiated by stepwise deesterification to form phthalate monoester and then PA sequentially [39]. Table 1 presents the catalysis profile of esterase activity of three Cyanobacteria during degradation of DMP. It was found that the presence of DMP resulted in the induction of esterases. Surprisingly, the esterase showed low activity in the culture right from the beginning at 50 mg L^−1^ DMP concentration; more interestingly, the activity was detected at 72 hours in the control as well. The esterase activity showed an increasing trend with increasing of incubation time and production of esterase by Cyanobacteria aided in the biodegradation of DMP. It was reported that deesterification is the first step in process of the DMP degradation of the* Bacillus* species, and two of the four isoesterases had very good DMP-hydrolyzing ability [40]. The esterase activity induced by DMP kept on increasing during the early period of degradation. This result also demonstrates that deesterification could be the initial step in the DMP degradation in Cyanobacteria.

*Synechocystis* sp. PCC 6803 is the first sequenced cyanobacterium [41]; subsequently, more cyanobacterial genome sequence was completed. The genome sequences of the three species of Cyanobacteria in this research are available (https://www.ncbi.nlm.nih.gov/). Five putative esterase genes were found in* Synechocystis, sll0644*,* slr1916*,* sll0992*,* sll1284,* and* slr8023*. Except for the defined esterase, four genes encoding probable esterases with the lotus tags synpcc7942_0451, synpcc7942_0458, synpcc7942_0774, and synpcc7942_2527 were detected in* Synechococcus* sp. PCC7942 as well as 3 genes with lotus tags Cyan7822_1061, Cyan7822_2840, and Cyan7822_3463 in* Cyanothece* sp. PCC7822. However, the expression and the functional esterase are remaining for further study.

## 4. Conclusions

Low concentration 20 mg L^−1^ DMP stimulated the growth of the three freshwater unicellular Cyanobacteria, but higher concentration could inhibited the growth. The Cyanobacteria investigated were capable of degrading DMP efficiently and* Cyanothece* was found to be the most effective species. PA as an intermediate degradation product of DMP accumulated in the Cyanobacteria culture and consequently caused the deduction of pH. 30°C is optimum for the DMP degradation of Cyanobacteria. It was observed that only a small portion of DMP aggregated into the cells; majority still remained in the medium. Esterase was induced by DMP and the activity kept increasing during the degradation. Taken together, the results of the study demonstrate that the three model Cyanobacteria can be applied into DMP degradation in aquatic system. More importantly, this study also provides basic knowledge to study the degradation mechanism in the molecular level further.

## Supplementary Material

HPLC of DMP and its degradation metabolites MMP and PA by Cyanothce sp. PCC7822 was shown in Figure S1, the degrading intermediates standardized by references.

## Figures and Tables

**Table 1:** Esterase activity profiles for cyanobacteria *Syenchocystis *sp. PCC6803 (6803), *Synechococcous* sp. PCC7942 (7942), and *Cyanothece* sp. PCC7822 (7822) at different incubation time, with 50 mg L^−1^ DMP (+DMP) or without DMP (−DMP).

Organism	+DMP			−DMP
	0 h	24 h	72 h	72 h
6803	0.06 ± 0.01	0.27 ± 0.03	0.96 ± 0.11	0.09 ± 0.01
7942	0.06 ± 0.01	0.26 ± 0.04	0.87 ± 0.06	0.13 ± 0.02
7822	0.07 ± 0.02	0.35 ± 0.03	1.11 ± 0.09	0.10 ± 0.02
