# Supplementary material for: Biodegradation of Dimethyl Phthalate by Freshwater Unicellular Cyanobacteria
Source: Biomed Res Int. 2016 Dec 19;2016:5178697. doi: 10.1155/2016/5178697 (PMC5204096; doi:10.1155/2016/5178697)
Supplement: Supplementary file 1 — HPLC of DMP and its degradation metabolites MMP and PA by Cyanothce sp. PCC7822 was shown in Figure S1, the degrading intermediates standardized by references. [file 5178697.f1.pdf]

Figure S1. High performance liquid chromatography of DMP and its degradation metabolites MMP and PA by *Cyanothce* sp. PCC7822 showing the degrading intermediates standardized by references.

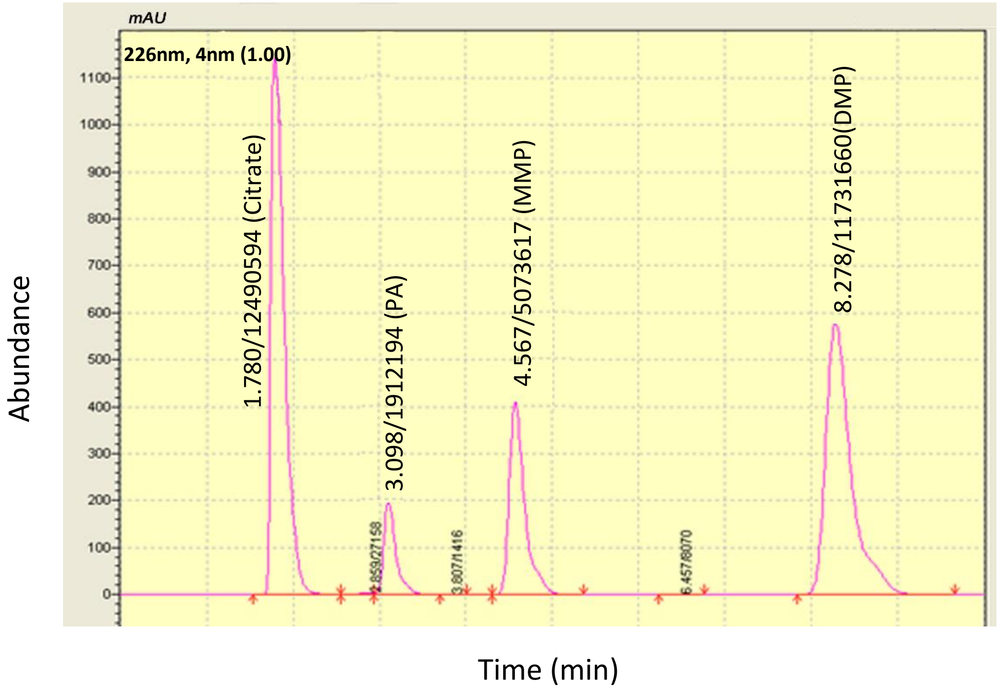

Figure S1
